# Comparison of Bioactive Secondary Metabolites and Cytotoxicity of Extracts from *Inonotus obliquus* Isolates from Different Host Species

**DOI:** 10.3390/molecules28134907

**Published:** 2023-06-22

**Authors:** Katarzyna Sułkowska-Ziaja, Justyna Robak, Andrzej Szczepkowski, Agnieszka Gunia-Krzyżak, Justyna Popiół, Joanna Piotrowska, Bartłomiej Rospond, Agnieszka Szewczyk, Katarzyna Kała, Bożena Muszyńska

**Affiliations:** 1 Department of Pharmaceutical Botany, Faculty of Pharmacy, Jagiellonian University Medical College, Medyczna 9, 30-688 Kraków, Poland; justyna.robak@student.uj.edu.pl (J.R.); agnieszka.szewczyk@uj.edu.pl (A.S.); k.kala@uj.edu.pl (K.K.); bozena.muszynska@uj.edu.pl (B.M.); 2 Institute of Forest Sciences, Department of Forest Protection, Warsaw University of Life Sciences—SGGW, Nowoursynowska 159, 02-776 Warszawa, Poland; andrzej_szczepkowski@sggw.edu.pl; 3 Department of Bioorganic Chemistry, Chair of Organic Chemistry, Faculty of Pharmacy, Jagiellonian University Medical College, Medyczna 9, 30-688 Kraków, Poland; agnieszka.gunia@uj.edu.pl; 4 Department of Pharmaceutical Biochemistry, Faculty of Pharmacy, Jagiellonian University Medical College, Medyczna 9, 30-688 Kraków, Poland; justyna.popiol@uj.edu.pl; 5 Department of Inorganic and Analytical Chemistry, Faculty of Pharmacy, Jagiellonian University Medical College, Medyczna 9, 30-688 Kraków, Poland; joanna.piotrowska@uj.edu.pl (J.P.); bartlomiej.rospond@uj.edu.pl (B.R.)

**Keywords:** *Alnus glutinosa*, Basidiomycota, *Betula pendula*, bioelements, *Carpinus betulus*, chaga, conk, medicinal mushrooms, mycelial cultures, triterpenes

## Abstract

*Inonotus obliquus*, a wood-decaying mushroom, has been used as a health-promoting supplement and nutraceutical for centuries. It is a source of bioactive compounds accumulated in both the conks (pseudosclerotia/sclerotia) and the biomass obtained in vitro. This study aimed to qualitatively and quantitatively analyze the bioelements and selected metabolites produced in mycelial cultures obtained from different host species. The mycochemical potential of mycelial cultures isolated from pseudosclerotia grown in *Betula pendula*, *Alnus glutinosa*, and *Carpinus betulus* was compared. Parent cultures were obtained in two types of medium (malt extract agar substrates without and with birch wood). Experimental cultures were developed in 2 L bioreactors for 10 days. The content of bioelements was determined using FAAS and FAES methods. Organic compounds were estimated using the RP–HPLC–DAD method. The cytotoxicity of the extracts was evaluated in human keratinocytes HaCaT, human skin fibroblasts BJ, human liver cancer HepG2, human melanoma A375, and mouse melanoma B16-F10. The extracts showed the presence of bioelements: sodium, potassium, magnesium, calcium, zinc, manganese, iron, and copper; phenolic acids: *p*-hydroxybenzoic, caffeic, *p*-coumaric, and protocatechuic; sterols: lanosterol, ergosterol, ergosterol peroxide; triterpene compounds: betulin, betulinic acid, inotodiol; indole compounds: L-tryptophan, tryptamine, 5-methyltryptamine, melatonin. The content of bioactive substances in the biomass was dependent on both the origin of the host species of the fungus isolate and the type of culture medium. Based on the results of this study, mycelial cultures can be proposed as a potential source of bioactive compounds and are promising naturally derived cytotoxic agents.

## 1. Introduction

For centuries, mushrooms have been used as therapeutic raw materials in traditional medical systems. Modern scientific research confirms that sporocarps are valuable sources of bioactive compounds, with a broad spectrum of medicinal properties.

As early as the sixteenth century, *Inonotus obliquus* (Fr.) Pilát, commonly known as Chaga, was used as an effective folk medicine in Russia and northern Europe to treat cancer, gastritis, ulcers, and tuberculosis [1,2] without causing toxic side effects [3].

The first descriptions of its use for medicinal purposes date back to 1880. Powdered chaga in the form of infusions was used to prevent and treat heart, liver, and stomach diseases, as well as tuberculosis [4]. Recent studies discuss the multidirectional effects of secondary metabolites isolated from *I. obliquus*. The anticancer, antimutagenic, cytostatic, antioxidant, antiviral, antihyperglycemic activities, etc., of extracts and individual compounds isolated from its conks have been demonstrated [5,6,7,8,9,10]. Furthermore, it has been confirmed that *I. obliquus* synthesizes more than 200 chemical compounds, including polysaccharides, triterpenoids, polyphenols, and steroids [11,12].

One of the alternative methods of traditional forest management, especially in low-production birch and alder stands, may be the cultivation of *I. obliquus* [13]. Therefore, knowledge of the content of bioactive compounds in cones from different geographic populations of *I. obliquus* and different hosts can provide useful information for this type of activity. Research may also lead to the discovery of more suitable, effective strains, i.e., strains better adapted to local conditions and specific host species and strains with a relatively high content of bioactive components.

The latest research, conducted by Adamson et al., provided interesting results regarding the effective cultivation of the natural resource *I. obliquus* [14].

A useful way to obtain bioactive metabolites derived from mushrooms is developing mycelial cultures. In some cases, this is the only method to obtain biological material for the research on and isolation of bioactive compounds while considering the protection of the species, especially if the species is rare. These methods are also useful in obtaining material for studies if the species is difficult to obtain from natural sites. Such difficulties may be attributable to geographic location or, as in the case of *I. obliquus*, a peculiar developmental cycle [15,16,17].

*I. obliquus* produces pseudosclerotia/sclerotia (conks, anamorphs) in the asexual stage and basidiomata (fruiting bodies) in the sexual stage (teleomorphs). Its basidiomata form in the outer layers of the wood adjacent to the pseudosclerotia, developing between the wood and the bark, and become visible only after the bark of the dead wood is cracked. These basidiomata are annual, resupinate, crustose, and impermanent. They reach a thickness of 0.5–2 cm, widths of several tens of centimeters, and lengths of up to several meters. Young basidiomata have a soft and corky texture, which becomes hard, brittle, and cracked with age. Unlike basidiomata, pseudosclerotia are perennial and can develop in the trunk of a living tree over several decades. They develop as a black growth with a cracked surface, as if charred, and can reach up to 50 cm in diameter, with a weight of more than 10 kg [18,19].

Pseudosclerotia *I. obliquus* contains various bioactive compounds, but quantitative and comparative studies of these compounds in cones on different host species are limited. Only previously published results of a comparative analysis of several bioactive compounds in the pseudosclerotia of *I. obliquus* with *Alnus incana*, *A. glutinosa* and *Betula pendula* from Northern Europe showed differences in the content of some compounds (e.g., betulinic acid) between tree species (*Alnus* v. *Betula*) and significant differences in the content of some compounds (e.g., inotodiol and lanosterol) in samples from the same tree species [20]. This indicates the need to test more strains of *I. obliquus* from different geographical locations for their bioactive compounds content. In our research, in addition to birch and alder strains, we used the *Carpinus betulus* strain from Central Europe. To the best of our knowledge, this study is the first study on the chemical makeup of *I. obliquus* parasitising *Carpinus betulus*. For the first time, a comparative analysis of the content of selected macro- and microelements in the obtained biomass was carried out, and nonhallucinogenic indole derivatives were also determined.

The aim of our research was to compare the chemical profile and cytotoxic potential of isolates obtained from three different species: *Betula pendula*, *Alnus glutinosa* and *Carpinus betulus*.

For this purpose, a qualitative and quantitative analysis of bioactive secondary metabolites and bioelements present in the biomass obtained from mycelial cultures of *I. obliquus* isolated from pseudosclerotia from various host species was carried out.

## 2. Results and Discussion

### 2.1. Evaluation of the Biotechnological Stage—Analysis of Biomass Increments

In the first stage of the experiment, the biomass gains obtained in an aerated culture during a 10-day culture cycle were estimated. While developing three independent cultures, the following average values biomass growths were obtained: for the isolate obtained from the pseudosclerotium grown in *C. betulus* (4 am), the dry mass (DM) weight of lyophilized mycelium obtained from 1 L of the liquid medium was 7.8 g (Table 1). For *B. pendula* isolates grown on malt extract agar (MEA) with the addition of birch wood (29 ambz and 30 ambz) and without this addition (29 am and 30 am), the average weight was 7.4, 9.9, 11.2, and 9.9 g/L DM, respectively. The weight of the mycelium of isolates grown in *A. glutinosa* (43 am and 76 am) was 2.8 and 4.8 g/L DM, respectively. In the case of isolates obtained from the pseudosclerotium overgrowing the *B. pendula*, regardless of the addition of birch wood, similar values of biomass increments were observed during 10-day culture cycles. In short, the highest increment (11.2 g/L DM) was observed for the isolate obtained from *B. pendula* and grown on MEA medium (30 am). The increment in biomass was about eight times higher compared with that of the inoculate. The lowest increase (2.8 g/L DM) was observed in the *A. glutinosa* isolate (43 am), which showed a twofold biomass increase compared with the inoculate (Table 1).

The rate and extent of mycelial growth in mycelial cultures depends on numerous factors, including the type of fungal strain that was used, the composition of the culture medium or the conditions of the culture environment.

The major host of *I. obliquus* is various species of *Betula* sp.; *Alnus* sp., *Fagus* sp., *Acer* sp., *Populus* sp. among others, can also act as its host [2,17]. Thus, this study attempted a comparison of the biosynthetic potential of the biomass depending on the host species. Understanding and controlling mycelial growth in submerged culture is important for the efficient and cost-effective production of fungal-derived products in industry [15]. An earlier study by Drenkhan et al. included a comparative analysis of the bioactive content of *I. obliquus* from *Alnus incana*, *A. glutinosa*, and *B. pendula* in Estonia and Finland. The authors focused on comparative mycochemical analysis [20]. In this study, the host spectrum was extended to include the *C. betulus* species and the focus was on comparing biomass increments and cytotoxic activity.

### 2.2. Assessing the Chemical Potential of Mycelial Cultures

Wood-decaying species such as *I. obliquus* can biosynthesize biologically active secondary metabolites, which are present in both biomass and culture media. Mycelial cultures represent a promising alternative for obtaining valuable therapeutic compounds. Using biotechnological methods to culture medicinal fungi significantly reduces the time to obtain biomass and allows for reproducible conditions to be used, resulting in standardized mycelium. At present, a few companies are using mycelial cultures to produce commercially available preparations. Since many fungi have huge potential to produce active compounds by cultures, a potential which is not yet fully understood, new substances with potential applications in medicine can be discovered by conducting research focusing on the identification of metabolites isolated from mycelial cultures [15,16].

Following previous studies on the wood-decomposition ability of various *I. obliquus* isolates [19], this study was conducted to determine the content of bioactive secondary metabolites in mycelium cultures obtained from various host species. In this study, the chemical profile and cytotoxic activity of the obtained biomass were determined.

### 2.3. Analysis of the Micro- and Macroelements

The contents of selected micro- and macroelements in the samples are shown in Table 2.

The presence of macroelements such as potassium, sodium, calcium and magnesium was observed in samples from all analyzed isolates/strains. Potassium was the dominant element. The highest potassium content (841.0 mg/100 g DM) was observed in the isolate/strain obtained from the pseudosclerotium growing on *A. glutinosa* (43 am), whereas the lowest potassium content (540.1 mg/100 g DM) was observed in the isolate/strain obtained from *B. pendula* with the addition of birch wood (29 ambz). For other isolates obtained from *B. pendula* (29 am, 30 am, and 30 ambz), the potassium content varied, which could be due to the storage method and pure culture, as well as the time needed to collect pseudosclerotia. A similar scenario was also observed in the isolate/strain obtained from *A. glutinosa*. In this case, the potassium content was lower, at 76 am (583.8 mg/100 g DM) than that of the 43 am sample obtained from the same tree species, which could be due to the difference in the time the pseudosclerotia were collected and the storage time of pure cultures (43 am was isolated in May 2011, whereas 76 am was isolated in September 2021). The sodium, calcium, and magnesium contents did not exceed 100 mg/100 g DM. The highest calcium content was observed in 30 am (92.8 mg/100 g DM), whereas the lowest was observed in 29 ambz (23.9 mg/100 g DM). The highest magnesium content was observed in 30 ambz, 37.0 mg/100 g DM. The following microelements were identified in mycelial cultures of *I. obliquus*: iron, zinc, manganese, and copper. Among these microelements, iron was the predominant one in isolates extracted from *C. betulus* (4 am), *B. pendula* (30 ambz), and *A. glutinosa* (43 am and 76 am), with a content of 31.0, 34.3, 32.6, and 29.4 mg/100 g of DM, respectively. The zinc content ranged from 3.2 to 11.3 mg/100 g DM, and the manganese content ranged from 3.1 to 10.8 mg/100 g DM.

Copper was determined in lower amounts in all isolates/strains except for 30 am. The highest iron (34.3 mg/100 g DM) and manganese (10.9 mg/100 g DM) contents were observed in *I. obliquus* isolated from pseudosclerotium growing on *B. pendula* in a tree line along a road in Warsaw and stored on malt extract agar (MEA, Carl Roth, Karlsruhe, Germany) with birch wood (30 ambz). The highest zinc content (11.3 mg/100 g DM) was observed in the isolate obtained from pseudosclerotium grown on *A. glutinosa* (43 am). Among all tested samples, *I. obliquus* isolated in May 2011 from *A. glutinosa* was characterized by an increased ability to accumulate potassium and zinc. A high accumulation potential for sodium and magnesium macroelements, as well as for iron and manganese microelements, was observed in mycelial cultures of *I. obliquus* extracted from pseudosclerotium grown on a *B. pendula* cultured on MEA medium with the 5% addition of birch wood (Table 2).

The ability of the sporocarps to accumulate bioelements is widely documented. Bioelements are components of enzymes or their activators [21,22,23].

To the best of our knowledge, the bioelements detected in the present study have not been the subject of analyses comparing the biomass obtained from different host species to date. The available data on the occurrence of these macro- and microelements in *I. obliquus* refer only to their presence in aqueous extracts. The results showed the high content of potassium and magnesium in the extract compared with the low content of sodium and calcium [24]. Other elements were found in trace amounts [25].

Our previous studies on the biolements content in arboreal species confirmed the presence of bioelements in both basidiomata and mycelial cultures. We examined and determined the macro- and microelement contents of *Ganoderma* sp., *Pleurotus* sp., and *Fomitopsis officinalis*, using both absorption and flame atomization emission spectrometry [26,27,28]. Of the tested species, namely *G. adspersum*, *G. applanatum*, *G. carnosum*, *G. lucidum*, *G. pfeifferi*, and *G. resinaceum*, mycelial cultures of *G. applanatum* showed the highest macroelement content, of potassium (1636.7 mg/100 g DM), sodium (1525.7 mg/100 g DM), calcium (387.8 mg/100 g DM), and magnesium (364.8 mg/100 g DM), and the highest microelement contents: manganese (53.2 mg/100 g DM), iron (63.3 mg/100 g DM), and zinc (20.1 mg/100 g DM). The highest copper content (1.1 mg/100 g DM) was observed in *G. pfeifferi* [26].

In addition to assessing the content of bioelements, our previous studie evaluated the effect of the addition of bioelements on their accumulation in the biomass. These studies show that the addition of zinc salts (zinc hydrogen aspartate, zinc sulfate (VI)) significally affected the individual macro- and microelement contents in the obtained biomass. Studies conducted on *Pleurotus* spp. confirm that a culture medium enriched with zinc salts contributes to the increased zinc accumulation in mycelial cultures (up to 193.4 mg/100 g DM). This shows that mycelial cultures are a better source of bioelements than sporocarps [29]. This trend was observed in *F. officinalis* [30].

To summarize, based on its substance accumulation capacity, mycelium of *I. obliquus* is a natural source of bioelements, with the potential to prevent deficiencies in these compounds.

### 2.4. Estimation of Phenolic Acids

A qualitative and quantitative analysis of phenolic acids was carried out, and the results are shown in Table 3.

Four phenolic acids were identified in methanolic extracts: *p*-hydroxybenzoic acid, protocatechuic acid, caffeic acid, and *p*-coumaric acid. Protocatechuic acid and *p*-hydroxybenzoic acid were observed in extracts of all isolates/strains. The highest *p*-hydroxybenzoic and protocatechuic acid content was observed in the isolate extract obtained from *B. pendula* grown on an MEA medium (29 am) 57.7 and 87.6 mg/100 g DM, respectively.

**Figure 1:**
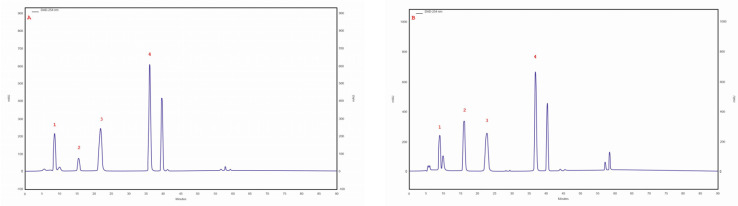
(**A**) sample chromatogram of the standards of selected phenolic acids: 1. protocatechuic acid, 2. *p*-hydroxybenzoic acid, 3. caffeic acid, 4. *p*-coumaric acid; (**B**) sample chromatogram of the extract from *Inonotus obliquus* mycelial cultures (isolate 29 am): 1. protocatechuic acid, 2. *p*-hydroxybenzoic acid, 3. caffeic acid, 4. *p*-coumaric acid.

The presence of caffeic and *p*-coumaric acid was observed in isolates/strains extracted from pseudosclerotium collected from *B. pendula* grown on an MEA medium with a 5% addition of birch wood (29 ambz, 30 ambz) and without this addition (29 am, 30ambz), and in isolates of 43 am ranging from 43.6 mg/100 g DM in 29 ambz (*p*-coumaric acid) to 3.2 mg/100 g DM in 43 am (caffeic acid). The chromatogram of the standards of the selected phenolic acids and the sample chromatogram of the extract from *I. obliquus* mycelium are shown in Figure 1.

Phenolic acids are compounds commonly found in sporocarps. The most common acids are gallic acid, caffeic acid, ferulic acid, protocatechuic acid, and vanillic acid. Syringic acid, ellagic acid, and chlorogenic acid are much less common [31].

Protocatechuic acid has proven antifungal, anti-inflammatory, antioxidant, chemopreventive, hepatoprotective, and neuroprotective activities; caffeic acid has antioxidant, chemopreventive, anticancer, hepatoprotective, and antimicrobial activities; *p*-coumaric acid has antimicrobial activities; and *p*-hydroxybenzoic acid has antimicrobial activities [32].

Recently, the phenolic acid content in *I. obliquus* and its biological activity have attracted the interest of many researchers. Studies have shown that both ethanol extracts and water extracts obtained from *I. obliquus* are characterized by the presence of phenolic compounds. Similar to the results obtained for the methanolic extract, protocatechuic acid and *p*-hydroxybenzoic acid have been identified, as well as gallic acid [16].

Glamoclija et al. reported interesting results in their study on *I. obliquus* extracts from Finland, Russia, and Thailand. They showed that the aqueous and ethanolic extracts of *I. obliquus* obtained from three different locations demonstrate quantitatively different compositions of phenolic acids [33].

Phenolic compounds, including phenolic acids, have also been determined in several species of polypores fungi. Protocatechuic acid, *p*-hydroxybenzoic acid, and vanillic acid have been identified in extracts from the basidiomata of *Daedaleopsis confragosa*, *Fomitopsis pinicola*, *Laetiporus sulphureus*, and *Fomitopsis betulina* (*Piptoporus betulinus*). Protocatechuic acid is the dominant phenolic acid, with values reaching the range of 17.7–90.0 µg/g dry weight. The highest phenolic acid content (114.9 µg/g DM) was observed in *F. pinicola* [34].

These results confirm the validity of the application of mycelial cultures, as mycelial cultures can be developed under repeatable conditions, thus resulting in a stable biomass composition. This facilitates the standardization of preparations derived from fungi for pharmaceutical use, for example. In addition, this technology could contribute to the protection of rare fungi in nature.

### 2.5. Estimation of Sterol Compounds

RP-HPLC-DAD analysis showed the presence of three sterols, namely ergosterol, ergosterol peroxide, and lanosterol, in extracts.

**Figure 2:**
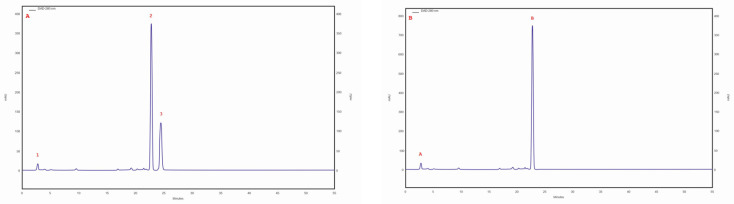
(**A**) sample chromatogram of the standards of selected sterol compounds: 1. lanosterol, 2. ergosterol, 3. ergosterol peroxide; (**B**) sample chromatogram of the extract from *Inonotus obliquus* mycelial cultures (isolate 4 am): 1. lanosterol, 2. ergosterol.

The ergosterol content was in the range of 223.5–99.5 mg/100 g DM. The highest ergosterol content was observed in the isolate/strain extracted from a *C. betulus* grown on MEA medium (4 am), whereas the lowest content was determined for the isolate/strain extracted from *B. pendula* and grown on MEA medium without the addition of birch wood (29 am). Furthermore, the lanosterol content was observed in all tested samples. The highest lanosterol content (129.2 mg/100 g DM) was observed in the methanolic extract obtained from an isolate/strain from *B. pendula* grown on MEA medium with a 5% addition of birth wood (30 ambz). In the remaining samples (94 am, 29 am, 29 ambz, 30 am, 43 am, and 76 am), it ranged from 94.8 to 68.1 mg/100 g of DM (Table 3). Trace amounts of ergosterol peroxide were observed in all studied samples. The chromatogram of the standards of the selected sterols and the sample chromatogram of the extract from *I. obliquus* mycelium are shown in Figure 2.

Sterols are a common group of compounds present in fungi. Sporocarps found nat-urally in the environment accumulate large amounts of sterols, whereas in mycelial cultures, these compounds accumulate to a lesser extent. In fungi, ergosterol plays a vital role in maintaining cell membrane fluidity and stability. It also serves as a pre-cursor for the synthesis of other important molecules, such as vitamin D_2_, which is produced by irradiating ergosterol with ultraviolet light [35].

Ergosterol is an important target for antifungal drugs, as many of them work by disrupting its synthesis or function. The presence or absence of ergosterol in fungal cells can also be used as a diagnostic tool to identify fungal infections. This has multidirectional pharmacological effects, including anticancer and immunostimulant activities, and is also a precursor of cortisol, an adrenal hormone with anti-inflammatory effects. Studies have shown that ergosterol and its peroxidation product, ergosterol peroxide, demonstrate pain-reducing effects associated with inflammation, reduce the incidence of cardiovascular disease, and inhibit cyclooxygenase [36]. Overall, ergosterol is a crucial molecule for the growth and survival of fungi, and understanding its role and regulation is important for the development of new antifungal treatments and could improve our understanding of fungal biology. Lanosterol (accounting for 45.47% of all sterols) and other sterol compounds, as well as intermediates of ergosterol synthesis such as 24-methylene, 4,4-dimethylfecosterol, fecosterol, and episterol, among others, were identified in naturally growing mycelia. Three sterols were determined in mycelial cultures, with ergosterol found to be dominant (82.20%) [37].

Methanolic extracts and aqueous extracts obtained from birch bark can induce the biosynthesis of steroidal compounds in *I. obliquus* cultures. RP-HPLC-DAD analysis showed that the extracts showed an improved biosynthesis of betulin, cholesterol, ergosterol, lanosterol, sitosterol, and stigmasterol. An aqueous extract of 0.01 g/L increases the production of steroids by up to 97.3% [38].

These results show that bioactive compounds are promising in *I. obliquus* grown not only on *Betula* sp. but also on *Alnus* sp. This favors the possibility of growing *I. obliquus* on *A. glutinosa* species, thus increasing the economic value of this tree species in forests.

### 2.6. Estimation of Triterpene Compounds

**Figure 3:**
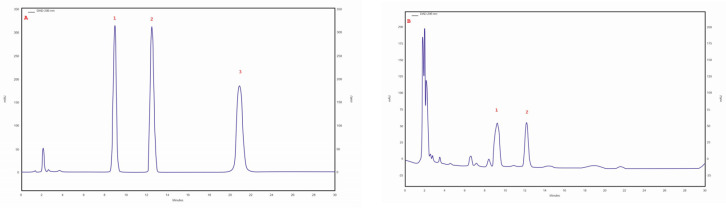
(**A**) sample chromatogram of the standards of selected triterpenes: 1. betulin, 2. betulinic acid, 3. inotodiol; (**B**) sample chromatogram of the extract from *Inonotus obliquus* mycelial cultures (isolate 30 am): 1. betulin, 2. betulinic acid.

Three triterpene compounds were identified in the mycelial cultures of all Chaga isolates: betulin, betulinic acid and inotodiol. The highest betulin (253.73 mg/100 g of DM) and betulinic acid (82.09 mg/100 g of DM) contents were determined in the biomass extract of the isolate/strain obtained from *B. pendula* (30 am) grown on an MEA medium. The betulin content for other isolates/strain was similar, ranging from 7.91 to 12.7 mg/100 g DM. Analyses showed that the lowest betulinic acid content (0.1 mg/100 g DM) was observed in the isolate of 43 am samples derived from pseudosclerotium collected from *A. glutinosa*. In all tested samples, trace amounts of inotodiol were identified (Table 3). The chromatogram of the standards of the selected triterpenes and the sample chromatogram of the extract from *I. obliquus* mycelium are shown in Figure 3.

The pentacyclic triterpenes present in *I. obliquus* are a therapeutically important group of compounds. They exhibit multidirectional pharmacological properties. Numerous chemical modifications of triterpene compounds can lead to the production of new derivatives with different biological activities [39]. There is no evidence to conclude that *I. obliquus* synthesizes betulin and betulinic acid de novo. Since birch bark is rich in these metabolites, the spinneret uses them for growth and development. Thus, their presence in mycelial cultures observed in the present study may result from the direct synthesis and transformation of previously produced cultures [40].

It is presumed that the presence of betulin and betulinic acid in Chaga is attributable to the association between the parasitic fungus and its plant host—in this case, the birch, or more specifically, the bark of the birch. In addition to the host type, environmental factors, geographic location, preservation techniques, and the time interval between harvesting and extract production can also affect the quantitative and qualitative composition of the metabolites [41].

Betulin is known for its antioxidant, antifungal, and antiviral activities. It has numerous medicinal properties, including anti-inflammatory, hepatoprotective, and antihyperuricemia activities. Betulin positively affects melanogenesis and has a radiation-protective effect. In addition, it has a lipotropic effect, as it lowers lipid levels in the liver, adipose tissue, and blood. Research has shown that betulinic acid and other triterpenes found in *I. obliquus* can inhibit the growth of various cancer cell lines by inducing apoptosis (programmed cell death) and inhibiting angiogenesis (the formation of new blood vessels) [42,43]. Furthermore, betulin and betulinic acid have been found to possess antioxidant properties, which may be beneficial for protecting cells against oxidative damage caused by free radicals [42]. Overall, the presence of betulinic acid and other triterpenes in *I. obliquus* is believed to contribute to its potential health benefits, and ongoing research is exploring its potential therapeutic applications [43]. Due to its complex structure, betulin is an excellent substrate for the synthesis of new compounds. In addition, its carboxyl derivative, betulinic acid (3β-hydroxy-lup-20(29)-en-28-ic acid), exhibits anticancer, antimalarial, hepatoprotective, antiparasitic, antiviral, antibacterial, and antifungal activities. In addition, it promotes the synthesis of collagen and elastin, prevents skin flaccidity, and inhibits gastric acid secretion. As shown in a study, the subcortical spinneret has 18 transcript sequences involved in the synthesis of terpenoids, including a squalene synthase responsible for producing triterpene compounds [44]. By 2016, approximately 40 lanostane-type triterpenoids were isolated. In addition to lanostane derivatives, subcortical spinnerets contain lupane-type triterpenoids (lupeol, lupenone, and betulin) and oleanane (oleanolic acid), although in much smaller amounts [44,45,46].

Of particular note are inotodiol, tramethenolic acid, betulin, and betulinic acid. The first two are lanostane-type triterpenes, whereas the latter two are lupane-type triterpenes [47,48].

### 2.7. Estimation of Indole Compounds

**Figure 4:**
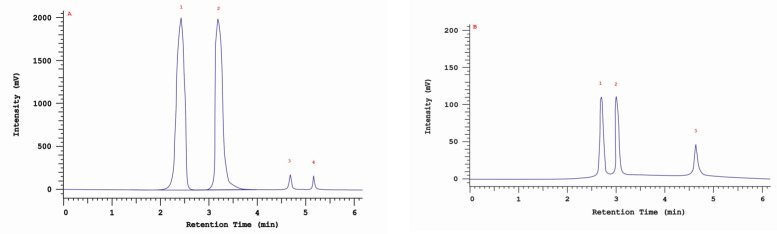
(**A**) Sample chromatogram of the standards of non-hallucinogenoc indole compounds: 1. L-tryptophan, 2. tryptamine, 3. methyltryptamine, 4. melatonin; (**B**) Sample chromatogram of the extract from *Inonotus obliquus* mycelial cultures (isolate 29ambz): 1. L-tryptophan, 2. tryptamine, 3. methyltryptamine.

An RP-HPLC-UV analysis showed the presence of four nonhallucinogenic indole compounds, L-tryptophan, tryptamine, 5-methyltryptamine and trace amounts of melatonin, in methanolic extracts. The presence of L-tryptophan was observed in all isolates/strains ranging from 30.4 mg/100 g DM (30 ambz) to 4.0 mg/100 g DM (43 am). The highest 5-methyltryptamine and L-tryptophan contents were observed in the extract obtained from isolate/strain of *I. obliquus* from *B. pendula* (29 am) grown on an MEA medium—5.5 mg/100 g DM. The presence of tryptamine was observed in five isolates/strains—4 am. 29 am, 29 ambz, 30 am and 30 ambz—which ranged from 0.8 to 2.5 mg/100 g DM (Table 3). The chromatogram of the standards of the selected indole compounds and the sample chromatogram of the extract from *I. obliquus* mycelium are shown in Figure 4.

Mushrooms are a rich source of precursors, neurotransmitters, and indole derivatives with confirmed antidepressant activity. Indole is a precursor for important chemical compounds found in nature, such as tryptophan, a naturally occurring essential amino acid that is broken down to tryptamine by bacteria. L-tryptophan is a precursor to 5-hydroxy-L-tryptophan, which is a direct substrate in the synthesis of serotonin. Serotonin is an indole compound with a hydroxyl group in the benzene ring. Indole compounds show significant pharmacological activity. Serotonin constricts blood vessels, plays an important role in directing brain impulses and is responsible for regulating sleep rhythms, aggression, anxiety, and body temperature. In addition, 5-hydroxy-L-tryptophan and L-tryptophan counteract the development of depression. Furthermore, melatonin controls the diurnal rhythm of physiological functions [49].

In fungi, melatonin is believed to play a role in the regulation of various physiological processes, including growth, development, and stress response. Studies have shown that melatonin production in fungi can be influenced by factors such as light, temperature, and nutrient availability. Melatonin can enhance the growth and development of some fungi, while also providing protection against oxidative stress. Other research has suggested that melatonin may also have antifungal properties and may be useful as a natural alternative to synthetic fungicides. Overall, the presence of melatonin in fungi suggests that it may have important physiological functions beyond its well-known role in regulating sleep and wakefulness in animals. Further research is needed to fully understand the role of melatonin in fungal biology and its potential applications in agriculture and medicine [49].

Our previous studies have confirmed the presence of indole compounds in biomass extracts from the in vitro cultures of *Fomitopsis betulina*, *F. officinalis*, or selected species of the genus *Ganoderma*, among others [28,50,51].

For example, the extracts from mycelial cultures of *F. betulina* showed the same qualitative composition as in the case of *I. obliquus*. The contents of individual compounds were as follows: L-tryptophan 1.3 mg/100 g DM, 5-hydroxy-L-tryptophan 2.8 mg/100 g DM, and 5-methyltryptamine 3.9 mg/100 g DM [50].

In the methanolic extract of *F. officinalis*, L-tryptophan, 6-methyl-D,L-tryptophan, melatonin, and 5-hydroxy-L-tryptophan were observed [28]. However, the content of individual compounds in the biomass of mycelial cultures in selected *Ganoderma* species varied, ranging from 10.6 mg/100 g DM (L-tryptophan in *G. lucidum*) to 0.01 mg/100 g DM (melatonin in *G. pfeifferi*) [51].

To our knowledge, the content of indole compounds in *I. obliquus* has not been extensively analyzed to date. This is the first study to confirm the 5-hydroxy-L-tryptophan, 5-methyltryptamine, and L-tryptophan contents in biomass from mycelial cultures.

### 2.8. Cytotoxicity Studies

**Figure 5:**
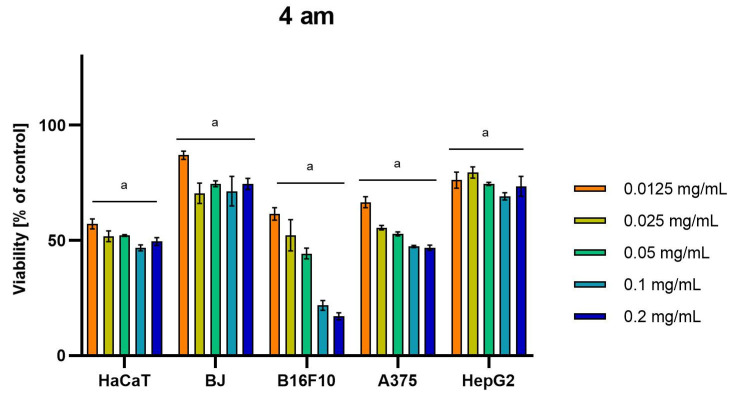
Results of cytotoxicity evaluation of 4 am (detailed characteristics of the strain are described in Table 4) in the concentration range of 0.0125–0.2 mg/mL. The viability was determined using an MTT assay. Each experiment was conducted in triplicate. Graphs represent the number of viable cells expressed as the percent of control ± SD. Results indicated with “a” were considered statistically significant (*p* < 0.05).

**Figure 6:**
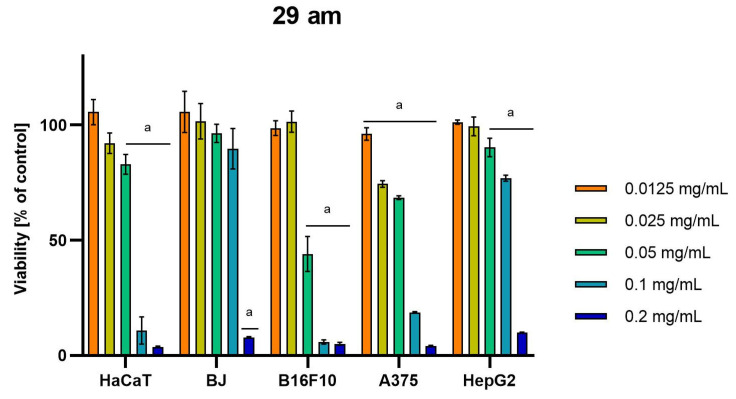
Results of cytotoxicity evaluation of 29 am (detailed characteristics of the strain are described in Table 4) in the concentration range of 0.0125–0.2 mg/mL. The viability was determined using an MTT assay. Each experiment was conducted in triplicate. Graphs represent the number of viable cells expressed as the percent of control ± SD. Results indicated with “a” were considered statistically significant (*p* < 0.05).

**Figure 7:**
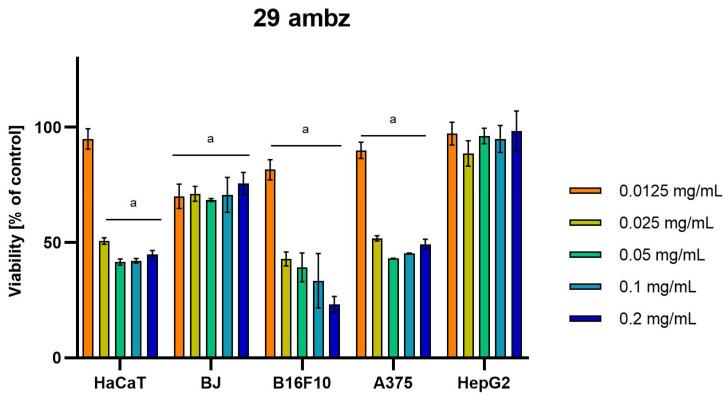
Results of cytotoxicity evaluation of 29 ambz (detailed characteristics of the strain are described in Table 4) in the concentration range of 0.0125–0.2 mg/mL. The viability was determined using an MTT assay. Each experiment was conducted in triplicate. Graphs represent the number of viable cells expressed as the percent of control ± SD.) Results indicated with “a” were considered statistically significant (*p* < 0.05).

**Figure 8:**
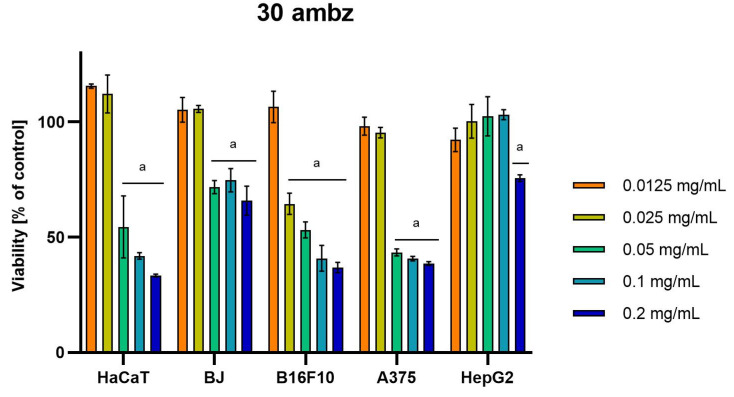
Results of cytotoxicity evaluation of 30 ambz (detailed characteristics of the strain are described in Table 4) in the concentration range of 0.0125–0.2 mg/mL. The viability was determined using an MTT assay. Each experiment was conducted in triplicate. Graphs represent the number of viable cells expressed as the percent of control ± SD. Results indicated with “a” were considered statistically significant (*p* < 0.05).

**Figure 9:**
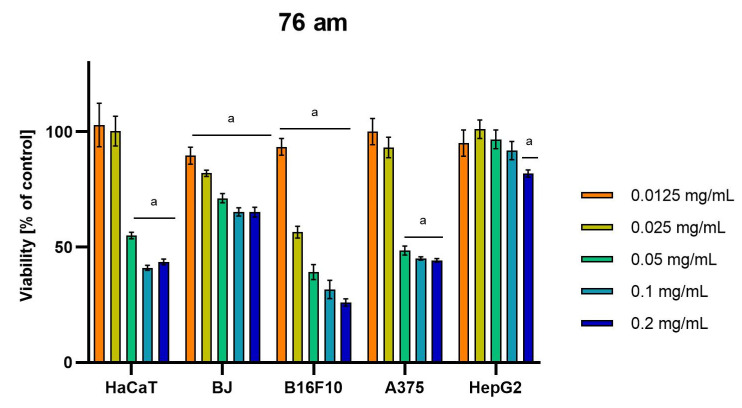
Results of cytotoxicity evaluation of 76 am (detailed characteristics of the strain are described in Table 4) in the concentration range of 0.0125–0.2 mg/mL. The viability was determined using an MTT assay. Each experiment was conducted in triplicate. Graphs represent the number of viable cells expressed as the percent of control ± SD. Results indicated with “a” were considered statistically significant (*p* < 0.05).

Differences were observed in the cytotoxic activity of the tested extracts; HepG2 was found to be the least sensitive cell line line (Figure 5, Figure 6, Figure 7, Figure 8 and Figure 9). Extracts 30 am and 43 am did not show cytotoxicity in any tested cell lines in the concentration range of 0.0125–0.2 mg/mL (except for B16F10 cells, in which 30 am caused a viability reduction of 34.5–37.7% in the concentrations of 0.1 and 0.2 mg/mL and 43 am of 38.1% in the concentration of 0.2 mg/mL. (Tables S1 and S2, Supplementary Materials.) The most interesting results were observed for extract 4 am (Figure 5), which caused a significant loss in viability in all tested cell lines and all tested concentrations, and for extract 29 am (Figure 6), which reduced viability by 90–96.2% in all tested cell lines at the highest tested concentration. Promising antimelanoma activity was observed in both human (A375) and mice (B16F10) cell lines for 4 am, 30 ambz, 29 ambz, 76 am, and 29 am (Figure 5, Figure 6, Figure 7, Figure 8 and Figure 9). However, these extracts were also cytotoxic to normal cell lines (HaCaT and BJ) (Figure 5, Figure 6, Figure 7, Figure 8 and Figure 9), so their application as active anticancer ingredients might be problematic. These results show that extracts obtained from *Carpinus betulus* and *Betula pendula* without the addition of wood birch are promising naturally derived cytotoxic agents, and further investigations in this area are needed.

Numerous studies confirm that the cytotoxic activity of Chaga extracts is associated with several active secondary metabolites [52]. Triterpene compounds such as betulin and betulinic acid have significant anticancer activity [41]. In the case of inotodiol, the cytotoxic activity was also proven. Thus, the compounds present in the studied extract may at least partially explain these effects. Cytotoxic effects were confirmed on various cell models [53]. Cytotoxic effects have been demonstrated, among others, on melanoma cells. Water extract not only inhibited the growth of B16-F10 cells by causing cell cycle arrest at G(0)/G(1) phase and apoptosis, but also induced cell differentiation [53]. Youn et al. proved that Chaga extract inhibited the human liver cancer HepG2 cell growth in a dose-dependent manner, which was accompanied by G0/G1-phase arrest and apoptotic cell death [54]. In addition, significant cytotoxic effects were confirmed for sarcoma cells and on A549 lung cells [55,56,57].

## 3. Materials and Methods

### 3.1. Sample Collection and Fungal Isolation to Mycelial Culture

The parent material for the study was *I. obliquus* isolates/strains from the collection of pure mycelial cultures obtained from the Department of Forest Protection, Institute of Forest Sciences, Warsaw University of Life Sciences (WAML-CK). *I. obliquus* isolates/strains were collected during the years 2007–2011 from the Polish population of this fungus. Pseudosclerotia, dense clusters of mycelia that encompass the substrate on which they grow and are necessary for the isolation of pure cultures, were obtained from the trunks of living *B. pendula*. *A. glutinosa*, and *C. betulus* trees. To obtain pure cultures, a small piece of mycelia from each pseudosclerotium was placed onto a 2% MEA (Malt Extract Agar, Carl Roth, Germany) at laminar chamber in laboratory. Mycelial cultures were carried out on two types of media: on MEA (4 am, 29 am, 30 am, 43 am, and 76 am) and on MEA with 5% birch wood (29 ambz and 30 ambz). Cultures were stored at about 5 °C in agar slants in glass tubes closed with a cotton wool plug and passed onto the newly prepared medium every 2 years. The characteristics of *I. obliquus* isolates are presented in Table 4.

### 3.2. Stationary Cultures

In the first stage of the study, stationary cultures were prepared on sterile agar medium in Petri dishes. The medium contained glucose (5%), yeast extract (1%), casein hydrolysate (1%), KH_2_PO_4_ (0.03%), and distilled water (up to 100%) at pH 6.5. A fragment of the isolate derived from the parental cultures was transferred to a 60 mm diameter glass Petri dish filled with solid agar medium. A Steri-Lite bead sterilizer (WPI, Worcester, MA, USA) was used during the passage. Twenty-eight dishes were prepared, four each for 4 am, 29 am, 29 ambz, 30 am, 30 ambz, 43 am, and 76 am samples. The plates were stored at ±22 °C under variable light conditions, maintaining a diurnal cycle. The cultures were passed every 3 weeks, each time using a newly prepared medium.

### 3.3. Submerged Cultures

During the next stage, submerged liquid cultures were established. Mycelial fragments (3 × 3 mm^3^) from a stationary culture of 3 weeks were transferred to 300 mL Erlenmeyer flasks containing 100 mL of sterile liquid medium. The flask outlet was protected with a 0.014 mm thick laboratory aluminum foil and parafilm. The flasks were placed on a rotary shaker with a frequency of 140 rpm (ALTEL, Łódź, Poland).

Twenty-one flasks were prepared, three from each sample series. The passage was carried out under a laminar air supply and horizontal flow of sterile air. Cultures were carried out at ±22 °C under variable light conditions, maintaining a diurnal cycle. After 3 weeks, the biomass passage was carried out in which 50 mL of the liquid culture was transferred to a freshly prepared medium.

### 3.4. Bioreactor Cultures

The final stage of the biotechnological process was conducting aerated cultures. To set these up, 2 L bioreactors (SIMAX^®^ glass bottles, Sázava, Czech Republic) containing 1.8 L of liquid sterile medium were used. The mycelium obtained from a 3-week culture conducted in Erlenmeyer flasks was transferred to the bioreactors. The biomass was constantly mixed using an aeration system, allowing for a constant supply of sterile air using filters (Millex60:5164218709GV Syringe Filter Unit, 0.22 µm, PVDF, 33 mm) and CO_2_ outflow using a Laboport^®^ mini-pump vacuum pump (KNF, Freiburg, Germany). The passage was carried out under laminar air supply in a horizontal sterile air flow box. Cultures were carried out at ±22 °C under variable light conditions in 10-day culture cycles. At the end of each cycle, the biomass was drained, washed with redistilled water, frozen, and freeze-dried in a freeze dryer (Labconco Corporation, Kansas City, MO, USA). For each isolate variant, three culture cycles were carried out.

Mycelial cultures were maintained in the medium containing glucose (5%), yeast extract (1%), casein hydrolysate (1%), KH_2_PO_4_ (0.03%), and distilled water (up to 100%) at pH 6.5. Culture plates with the medium were sterilized using an ASVE autoclave (Cooperative of Mechanical Workers, Warsaw, Poland) at 121 °C with an overpressure of 1 atm. All experiments were carried out in triplicate, which ensured that the observed trends were reproducible.

### 3.5. Estimation of Bioelements

The lyophilized biomass of the culture of each isolate was pulverized in an agate mortar, which weighed exactly 0.5 g in Teflon plates. This was followed by the addition of 2.0 mL of a 30% H_2_O_2_ solution and 4.0 mL of a 65% HNO_3_ solution.

In a Magnum II microwave apparatus (ERTEC-Poland, Wrocław, Poland), mineralization was carried out wet in a closed system. The mineralization process consisted of three stages, with each stage lasting 10 min with a microwave power of 80%, 90%, and 100%, respectively, at 290 °C, and a pressure of 45 bar in all stages. After mineralization was completed, the solutions were transferred to quartz evaporators and evaporated to an almost dry state at 150 °C using a hot plate. The residues were quantitatively transferred to 10 mL volumetric flasks with distilled water four times.

An iCE3500 spectrometer (Thermo Scientific, Gloucester, UK) was used in the quantitative analysis of selected trace elements (Mn, Zn, Cu, and Fe) and macroelements (Mg, Na, K, and Ca). Flame atomic absorption spectrometry (FAAS) was carried out to determine Mn, Zn, Cu, Fe, Mg, K, and Ca, whereas flame atomic emission spectrometry (FAES) was used to determine Na.

### 3.6. Estimation of Organic Compounds

The medium present in the biomass obtained after the culture cycle was drained, and the resulting biomass was rinsed with redistilled water, frozen, and then lyophilized. Then, 3.0 g of biomass was weighed and transferred to 15 mL polypropylene tubes, and 5 mL of HPLC-grade methanol was added. An extraction process was carried out (four cycles of 20 min each) using ultrasound with a frequency of 40 kHz (Sonic-3, Polsonic, Poland). The extracts were then centrifuged in a laboratory centrifuge apparatus (MPW-342, Warsaw, Poland) for 10 min at 4300 rpm. The resulting supernatant was transferred to glass crystallizers and left at room temperature until the evaporation of methanol. The procedure was repeated thrice. The dry residue was dissolved in 3 mL of HPLC-grade methanol, and then the contents of each sample were filtered through a 0.22 µm PTFE syringe filter into glass vials (Witko Sp. z o.o. Łódź, Poland). The extracts were stored in a refrigerator (2–8 °C) until HPLC analysis.

Methanolic extracts were used to determine phenolic acids, sterols, and indole compounds, whereas isopropyl alcohol extracts were used to determine triterpenes.

A reversed-phase, high-pressure liquid chromatography equipped with a diode array detector RP-HPLC-DAD method was used to analyze the content of organic compounds, phenolic acids, sterols, and triterpenes. The liquid chromatography setup included the following: an HPLC analyzer (Merck Hitachi, Tokyo, Japan), an L-2455 DAD detector, an L-2350 thermostat, an L-2130 pump, an end-capped column of LiChroCART^®^ 250-4 Purospher^®^ STAR RP-18 (5 µm), and an L-2200 autosampler. For the analysis of indoles, a setup containing an HPLC analyzer (Merck Hitachi, Tokyo, Japan), an L-7400 UV detector, an end-capped column of LiChroCART^®^ 250-4 Purospher^®^ STAR RP-18 (5 µm), an L-2350 thermostat, an L-7100 pump, and a VWR7614 degasser was used.

Phenolic acids were analyzed as previously described [58]. The mobile phase used for the gradient program consisted of two solvents: methanol with 0.5% acetic acid in a volume ratio of 1:4 (*v*/*v*). Calculations were performed at a wavelength of 254 nm. Sterols were analyzed as previously described [50,59]. The mobile phase used for the gradient program consisted of two solvents: methanol/water 80:20 (*v*/*v*) and methanol/dichloromethane 75:25 (*v*/*v*). Calculations were performed at a wavelength of 280 nm. Triterpenes were analyzed using a previously described method [51,60]. The mobile phase used for the isocratic system consisted of an acetonitrile/water mixture in a 9:1 *v*/*v* ratio. Calculations were performed at a wavelength of 206 nm. Nonhallucinogenic indole compounds were analyzed using a previously described method [61]. The mobile phase used for the isocratic system consisted of a methanol/water/0.1 M ammonium acetate mixture in a 15:14:1 *v*/*v* ratio. Calculations were performed at a wavelength of 280 nm.

Organic compounds were identified by compiling retention times and UV adsorption spectra obtained during the chromatographic analysis of the solutions of standard substances and the solutions of test substances. The data obtained from the chemical analysis were subjected to a one-way analysis of variance, followed by a post hoc Tukey test, using the STATISTICA v.13 software (Statsoft, Tulsa, OK, USA). All experiments were carried out in triplicate, and data were presented as mean ± standard deviation (SD). Furthermore, differences between groups were considered statistically significant when *p*-values were 0.05 or less.

### 3.7. Standard Substances and Reagents Used in the Analysis

The standard substances used in the analyses were purchased from the following manufacturers: indole and sterol compounds from Sigma-Aldrich (Darmstadt, Germany); phenolic acids from Sigma-Aldrich (Darmstadt, Germany) and Honeywell—Fluka TM (Buchs, Switzerland); triterpenes from Sigma-Aldrich (Darmstadt, Germany) and ChemFaces (Wuhan, China). Reagents used in the analyses were purchased from Honeywell-Riedel de HaenTM (Seelze, Germany) and Avantor Performance Materials Poland S.A (Gliwice, Poland).

### 3.8. Cytotoxicity Studies

Cytotoxicity of the extracts was evaluated on the following cell lines: human keratinocyte HaCaT (Sigma-Aldrich, Darmstadt, Germany), human skin fibroblast BJ (ATCC, Manassas, VA, USA), human liver cancer HepG2 (ATCC, Manassas, VA, USA), human melanoma A375 (ATCC, Manassas, VA, USA), and mouse melanoma B16-F10 (ATCC, Manassas, VA, USA). Cells were grown in culture flasks in an incubator under standard conditions (37 °C, CO_2_ concentration 5%). They were then cultured in an appropriate culture medium according to the manufacturer’s instructions, and supplemented with 10% fetal bovine serum (Gibco, Waltham, MA, USA) and antibiotics (Gibco, Waltham, MA, USA). The cells were seeded on 96-well plates at a density of 10,000/well for HaCaT, BJ, HepG2, and A375 and 5000/well for B16-F10. Then, they were incubated for 24 h, the medium was changed, and extracts were added to the wells at a final concentration of 0.0125–0.2 mg/mL. Dimethyl sulfoxide (DMSO) and pure medium were used as controls. After 48 h of incubation ((37 °C, 5% CO_2_), MTT (3-(4,5-dimethylthiazol-2-yl)-2,5-diphenyltetrazolium bromide, Sigma-Aldrich, Darmstadt, Germany) solution (5 mg/mL) was added to the medium. The formation of dark formazan crystals was observed at the bottom of the wells after 3–4 h of incubation; then, the medium was removed, and formazan was dissolved in DMSO. A multiwell plate reader (Spectra Max iD3, Molecular Devices, San Jose, CA, USA) was used to detect absorbance at 570 nm. Cell viability was determined by dividing the absorbance of the experimental wells by the absorbance of the vehicle control wells ×100%. The experiment was conducted in triplicate. Data were subjected to a one-way analysis of variance, followed by Dunnett’s test, using GraphPad Prism 9.0 software (GraphPad Software Inc., San Diego, CA, USA). Values of *p* < 0.05 were considered statistically significant. The results were expressed as means ± SD of the mean.

## 4. Conclusions

In this study, a comparison of the chemical composition and cytotoxic potential of biomass from mycelial cultures obtained from different host species was carried out. Based on the results, it can be concluded that both the host species from which mycelium was obtained and the type of culture medium affect the contents of bioactive substances in the obtained biomass. The highest content of the analyzed substances was characterized by mycelial cultures obtained from *Betula pendula*, whereas the lowest content was characterized by mycelial cultures obtained from *Alnus glutinosa*. The analyzed mycelial cultures can be proposed as potential sources of compounds with applications in the prevention of various diseases and can also be used in the supplementation of certain components.

The presence of metabolites with chemical and pharmacological diversity in *I. obliquus* shows that it is a favorable medicinal mushroom for drug discovery. However, due to the slow formation of mycelia in natural habitats, these pharmacologically im-portant metabolites cannot be produced for therapeutic purposes using naturally occurring material. Thus, mycelial cultures are a useful solution for the large-scale production of bioactive metabolites.

The results obtained in this study suggest that mycelial cultures of *I. obliquus* derived from different host species produce significant amounts of bioactive organic compounds, which, along with the labeled bioelements, play an important role in natural medicine, prevention of various diseases, and plant physiology.

## Figures and Tables

**Table 1:** Comparison of biomass increment values of experimental cultures of *Inonotus obliquus*.

Biomass Increment Values—DM in g/L of Medium							
Sample Name							
	4 am	29 am	29 ambz	30 am	30 ambz	43 am	76 am
M_0_	1.5	1.5	1.5	1.5	1.5	1.5	1.5
MM	7.8 ± 0.3 ^a^	7.4 ± 0.7 ^a^	9.9 ± 1.0 ^b^	11.2 ± 0.9 ^c^	9.9 ± 0.8 ^b^	2.8 ± 0.6 ^d^	4.8 ± 0.7 ^d^

*n* = 3; DM—dry mass; M_0_—mass of inoculate; MM—biomass after 10-day of growth cycle; detailed characteristics of individual isolates (4 am, 29 am, 29 ambz, 30 am, 30 ambz, 43 am, 76 am) are described in Table 4. The letters next to values represent Tukey’s HSD post hoc results (*p* < 0.05) according to the compact letter display convention, i.e., a letter refers to the group (a–d) in which there was no significant difference.

**Table 2:** Content of bioelements in biomass of *Inonotus obliquus* (mg/100 g DM).

Sample Name							
	4 am	29 am	29 ambz	30 am	30 ambz	43 am	76 am
Macroelements							
K	570.9 ± 14.1 ^acd^	798.8 ± 44.3 ^bcde^	540.1 ± 27.6 ^abcd^	558.1 ± 40.4 ^abcde^	616.4 ± 19.9 ^abcde^	841.0 ± 46.9 ^abde^	583.8 ± 6.9 ^abcde^
Na	9.1 ± 0.1 ^a^	5.6 ± 0.2 ^b^	5.8 ± 0.3 ^b^	13.9 ± 0.3 ^c^	15.6 ± 0.9 ^d^	8.7 ± 0.3 ^e^	11.8 ± 0.56 ^f^
Ca	27.9 ± 0.5 ^a^	24.8 ± 0.7 ^a^	23.9 ± 3.5 ^a^	92.8 ± 1.2 ^b^	88.0 ± 3.4 ^b^	37.1 ± 1.7 ^c^	49.8 ± 0.78 ^d^
Mg	16.2 ± 0.2 ^ade^	17.6 ± 0.3 ^ade^	20.3 ± 0.5 ^be^	21.9 ± 1.3 ^b^	37.0 ± 0.80 ^c^	17.8 ± 0.30 ^ade^	18.3 ± 1.04 ^abe^
Microelements							
Fe	31.0 ± 1.3 ^a^	7.1 ± 0.4 ^bcd^	9.5 ± 0.1 ^b^	8.1 ± 0.1 ^b^	34.3 ± 1.8 ^ac^	32.6 ± 2.3 ^acd^	29.4 ± 0.3 ^acd^
Zn	6.9 ± 0.7 ^ad^	6.2 ± 0.1 ^bd^	3.2 ± 0.1 ^c^	3.2 ± 0.1 ^c^	6.7 ± 0.1 ^abd^	11.3 ± 0.1 ^e^	7.8 ± 0.1 ^f^
Mn	4.4 ± 0.2 ^a^	7.3 ± 0.2 ^b^	8.6 ± 0.1 ^c^	7.9 ± 0.2 ^d^	10.3 ± 0.2 ^e^	3.1 ± 0.1 ^f^	5.5 ± 0.1 ^g^
Cu	0.1 ± 0.1 ^abc^	0.2 ± 0.1 ^abe^	0.1 ± 0.1 ^ac^	nd	0.0 ± 0.1 ^ac^	0.4 ± 0.1 ^de^	0.3 ± 0.1 ^de^

*n* = 3; detailed characteristics of individual isolates (4 am, 29 am, 29 ambz, 30 am, 30 ambz, 43 am, 76 am) are described in Table 4. The letters next to the values represent Tukey’s HSD post hoc results (*p* < 0.05) according to the compact letter display convention, i.e., a letter refers to the group (a–g) in which there was no significant difference.

**Table 3:** Qualitative and quantitative composition of the analyzed samples of *Inonotus obliquus* (mg/100 g DM).

Sample Name							
Analyzed Compounds	4 am	29 am	29 ambz	30 am	30 ambz	43 am	76 am
Phenolic Acids							
*p*-Hydroxybenzoic acid	12.5 ± 0.1 ^ac^	57.7 ± 0.1 ^b^	11.6 ± 0.7 ^ac^	20.3 ± 13.3 ^acd^	17.1 ± 0.1 ^acd^	29.0 ± 0.1 ^cd^	12.1 ± 0.5 ^ac^
Protocatechuic acid	8.6 ± 0.2 ^a^	87.6 ± 3.1 ^b^	41.7 ±3.6 ^c^	12.4 ± 0.1 ^a^	24.5 ± 0.1 ^d^	8.8 ± 0.1 ^a^	9.1 ± 0.1 ^a^
Caffeic acid	nd	8.6 ± 0.2 ^a^	3.8 ± 0.5 ^a^	37.0 ± 9.0 ^b^	4.0 ± 0.1 ^a^	3.2 ± 0.3 ^a^	nd
*p*-Coumaric acid	nd	43.6 ± 1.4 ^bc^	37.1 ± 2.3 ^bc^	29.3 ± 2.1 ^bc^	26.8 ± 0.7 ^bcd^	17.8 ± 0.8 ^acde^	nd
Sterols compounds							
Ergosterol	223.5 ± 0.1 ^a^	99.5 ± 2.3 ^b^	166.2 ± 2.6 ^cf^	155.0 ± 0.1 ^def^	146.1 ± 0.1 ^de^	160.9 ± 3.9 ^cdf^	150.8 ± 7.1 ^def^
Ergosterol peroxide	*	*	*	*	*	*	*
Lanosterol	76.1 ± 4.0 ^a^	74.4 ± 1.3 ^a^	94.8 ± 0.4 ^b^	45.8 ± 0.1 ^c^	129.2 ± 01 ^d^	89.1 ± 1.1 ^e^	68.1 ± 1.2 ^f^
Triterpenes							
Betulin	7.9 ± 0.1 ^a^	12.7 ± 0.2 ^b^	10.2 ± 0.3 ^cf^	253.7 ± 0.9 ^d^	9.1 ± 0.2 ^e^	10.5 ± 0.1 ^ce^	8.88 ± 0.1 ^e^
Betulinic acid	5.7 ± 0.6 ^ae^	18.7 ± 0.1 ^b^	16.9 ± 0.1 ^b^	82.1 ± 0.7 ^c^	16.3 ± 2.2 ^b^	0.1 ± 0.1 ^d^	4.8 ± 0.3 ^e^
Inotodiol	*	*	*	*	*	*	*
Non-hallucinogenic indole compounds							
L-tryptophan	13.1 ± 3.8 ^ac^	19.7 ± 0.3 ^bcd^	17.4 ± 2.4 ^abc^	22.9 ± 0.3 ^bd^	30.4 ± 1.2 ^e^	4.0 ± 0.7 ^f^	18.7 ± 2.0 ^bcd^
Tryptamine	0.9 ± 0.2 ^a^	0.5 ± 0.3 ^a^	2.5 ± 0.1 ^b^	1.8 ± 0.2 ^c^	0.8 ± 0.2 ^a^	nd	nd
5-Methyltryptamin	nd	5.5 ± 0.1 ^b^	3.2 ± 0.1 ^cd^	3.3 ± 0.1 ^cde^	3.4 ± 0.1 ^de^	nd	nd
Melatonin	nd	*	*	*	*	nd	nd

*n* = 3; DM—dry mass; *—trace amount; nd—not detected; detailed characteristics of individual isolates (4 am, 29 am, 29 ambz, 30 am, 30 ambz, 43 am, 76 am) are described in Table 4. The letters next to values represent Tukey’s HSD post hoc results (*p* < 0.05) according to the compact letter display convention, i.e., a letter refers to the group (a–f) in which there was no significant difference.

**Table 4:** Characteristics of *Inonotus obliquus* isolates/strains used in this study.

Sample Name	Isolate/Strain No in WAML-CK	Date of Isolation and Included in WAML-CK	Origin of Pseudosclerotia	Host Species	Medium/Passage Time
4 am	WAML-CK 4	XII 2007	Grójec Forest District	*Carpinus betulus*	MEA, passaged every 2 years
29 am	WAML-CK 29	XI 2008	Sarnaki Forest District	*Betula pendula*	MEA, passaged every 2 years
29 ambz	WAML-CK 29	XI 2008	Sarnaki Forest District	*B. pendula*	MEA, 5% birch wood addition, passaged every 2 years
30 am	WAML-CK 30	XII 2008	Warszawa Avenue/Dolina Służewiecka Str.	*B. pendula*	MEA, passaged every 2 years
30 ambz	WAML-CK 30	XII 2008	Warszawa Avenue/Dolina Służewiecka Str.	*B. pendula*	MEA, 5% birch wood addition, passaged every 2 years
43 am	WAML-CK 43	V 2011	Sarnaki Forest District	*Alnus glutinosa*	MEA, passaged every 2 years
76 am	WAML-CK 76	IX 2021	Pułtusk Forest District	*A. glutinosa*	MEA

Data from the collection of the Department of Forest Protection, Institute of Forest Sciences, Warsaw University of Life Sciences (compiled by Andrzej Szczepkowski).

## Data Availability

Not applicable.

## Supplementary Materials

The following supporting information can be downloaded at: https://www.mdpi.com/article/10.3390/molecules28134907/s1, Table S1: Results of cytotoxicity evaluation of 30am in concentration range of 0.0125–0.2 mg/mL. The viability was determined using MTT assay. Each experiment was repeated in triplicate. The results represent the number of viable cells expressed as a percent of the control ± SD; Table S2: Results of cytotoxicity evaluation of 43am in concentration range of 0.0125–0.2 mg/mL. The viability was determined using MTT assay. Each experiment was repeated in triplicate. Results represent the number of viable cells expressed as percent of control ± SD.
