# Supplementary material for: Comparison of Bioactive Secondary Metabolites and Cytotoxicity of Extracts from Inonotus obliquus Isolates from Different Host Species
Source: Molecules. 2023 Jun 22;28(13):4907. doi: 10.3390/molecules28134907 (PMC10343780; doi:10.3390/molecules28134907)
Supplement: Supplementary file 1 [file molecules-28-04907-s001.zip › molecules-2406303-supplementary.pdf]

Comparison of bioactive secondary metabolites and cytotoxicity of extracts from *Inonotus obliquus* isolates from different host species

Additional data on the cytotoxicity of extracts

Table S1. Results of cytotoxicity evaluation of 30am in concentration range of 0.0125 - 0.2 mg/mL. The viability was determined using MTT assay. Each experiment was repeated in triplicate. The results represent the number of viable cells expressed as a percent of the control ± SD

Concentration [mg/mL]	HaCaT		BJ		B16F10		A375		HepG2	
	viability	SD	viability	SD	viability	SD	viability	SD	viability	SD
0.0125	102.46	2.03	106.91	4.68	104.72	4.28	93.49	4.31	90.44	5.94
0.025	103.34	7.24	93.62	4.82	93.17	5.03	94.76	4.37	100.81	4.58
0.05	96.13	4.80	103.81	6.97	96.45	5.46	95.03	2.89	100.32	1.49
0.1	90.36	3.67	86.43	4.38	65.49	13.40	92.13	3.08	103.34	1.48
0.2	101.59	4.45	84.65	5.66	62.32	6.02	87.52	1.65	108.53	6.21

Table S2. Results of cytotoxicity evaluation of 43am in concentration range of 0.0125 - 0.2 mg/mL. The viability was determined using MTT assay. Each experiment was repeated in triplicate. Results represent the number of viable cells expressed as percent of control ± SD

Concentration [mg/mL]	HaCaT		BJ		B16F10		A375		HepG2	
	viability	SD	viability	SD	viability	SD	viability	SD	viability	SD
0.0125	105.66	2.67	94.90	4.70	102.33	9.00	104.41	0.76	93.93	1.26
0.025	94.99	1.84	96.59	2.49	103.73	5.21	101.30	2.86	102.24	4.78
0.05	102.98	2.96	92.07	2.63	109.98	14.52	97.16	4.25	101.07	2.97
0.1	97.36	5.51	92.83	2.52	105.28	14.37	99.44	2.63	108.64	5.22
0.2	104.03	6.49	92.86	2.24	61.90	10.20	94.28	0.98	102.24	2.92
